# Supplementary material for: A fault-intrusion-tolerant system and deadline-aware algorithm for scheduling scientific workflow in the cloud
Source: PeerJ Comput Sci. 2021 Nov 2;7:e747. doi: 10.7717/peerj-cs.747 (PMC8576554; doi:10.7717/peerj-cs.747)
Supplement: Supplemental Information 6 [file peerj-cs-07-747-s006.zip › Myproject1.1/docs/org/workflowsim/utils/package-use.html]

Uses of Package org.workflowsim.utils


JavaScript is disabled on your browser.


- Overview
- Package
- Class
- Use
- Tree
- Deprecated
- Index
- Help

- Prev
- Next

- Frames
- No Frames

- All Classes

# Uses of Package org.workflowsim.utils

- Packages that use org.workflowsim.utils

  | Package | Description |
  | --- | --- |
  |  |  |
  | --- | --- |
  | org.workflowsim.utils |  |
- Classes in org.workflowsim.utils used by org.workflowsim.utils

  Class and Description

  ClusteringParameters

  ClusteringParameters contains all the parameters used in task clustering

  ClusteringParameters.ClusteringMethod

  Supported Clustering Method, by default it is none

  OverheadParameters

  This class includes all parameters that involve overheads

  Parameters.FTCFailure

  Parameters.FTCMethod

  Fault Tolerant Clustering method

  Parameters.FTCMonitor

  Parameters.PLNMethod

  Planning Algorithm (Global Scheduling Algorithm)

  Parameters.SCHMethod

  ReplicaCatalog.FileSystem

  File System

- Overview
- Package
- Class
- Use
- Tree
- Deprecated
- Index
- Help

- Prev
- Next

- Frames
- No Frames

- All Classes
